# Polymerase chain reaction ribotyping of *Clostridium difficile*isolates in Qatar: a hospital-based study

**DOI:** 10.1186/1471-2334-14-502

**Published:** 2014-09-15

**Authors:** Asma A Al-Thani, Wedad S Hamdi, Naser A Al-Ansari, Sanjay H Doiphode

**Affiliations:** Virology Health Sciences Department, College of Arts and Sciences, Qatar University, Doha, Qatar; Department of Laboratory Medicine and Pathology, Al-Khor Hospital, Hamad Medical Corporation, Doha, Qatar

**Keywords:** Clostridium difficile, Ribotype, Qatar

## Abstract

**Background:**

*Clostridium difficile* infection (CDI) is not generally reported to public health authorities in the Middle East and its true prevalence remains largely unknown. The aims of this study were to determine the prevalence of CDI and its associated ribotypes among *C. difficile* isolates in Qatar. Influence of age and correlation with other risk factors e.g. proton pump inhibitor use, antibiotic use, existence of chronic conditions, etc was also investigated for CDI positive patients.

**Methods:**

A total of 1,532 patients with suspected CDI were recruited from two hospitals between 2011 and 2012. *C. difficile* was identified using glutamate dehydrogenase (GDH) lateral flow assay and toxins A and B Enzyme Immunoassay (EIA). The *C. difficile* positive samples were then cultured for PCR-ribotyping.

**Results:**

122 of the 1,532 (7.9%) samples from individual patients were identified as *C.difficile* positive; and 79 of these were viably cultured (~65%). From these, 36 different PCR ribotypes were isolated, of which strains 258 (6 [7.6%]), 01/014/046 (5 [6.3%]), and 011/053/056/107 (4 [5%]) were the most prevalent. The prevalence of PCR-ribotype 027 was 1.3% (n = 1). An age of ≥65 years and treatment with proton pump inhibitors correlated with higher frequency of CDI. Treatment with third generation cephalosporins (50 [41%]) and piperacillin/tazobactam antibiotics (55 [45.1%]) was most frequently associated with CDI.

**Conclusion:**

The most common *C. difficile* ribotype identified in Qatar was 258, which is different from those found in North America, Europe and Asia. The prevalence of CDI was higher in Qatar than Europe; though comparable to other Middle Eastern countries. These findings underscore the importance of local surveillance to detect and control *C. difficile* infection.

**Electronic supplementary material:**

The online version of this article (doi:10.1186/1471-2334-14-502) contains supplementary material, which is available to authorized users.

## Background

Infection with *Clostridium difficile* is a common cause of diarrhoea, especially diarrhoea associated with a history of antibiotic use [1]. *C. difficile* has been identified as the major pathogen implicated in nosocomial diarrhoea arising >72 hours after admission among patients receiving antibiotics [2]. The infection can range from mild diarrhoea to severe pseudomembranous colitis. Risk factors for *C. difficile* infection (CDI) include antibiotic exposure, hospitalisation, and advanced age [1].

The epidemiology and microbiology of CDI varies according to regional differences. In North America and Europe, the epidemiology of CDI is well-documented. For example, the clindamycin-resistant ribotype 017 strain of apparent clonal origin has been the cause of epidemics in Canada, the Netherlands, Ireland and Asia [3–7]. However, ribotypes 027 and 078, which are the main causes of outbreaks in other regions of the world, appear to be rare in Asian countries [7]. It is important to assess CDI in the Arab world, where limited data are available on both CDI prevalence and its ribotypes, despite over-prescription of antibiotics in this region [8–12].

In Qatar, CDI is not necessarily reported to the public health authorities and its true prevalence remains unknown. This may be due to the poor awareness of antibiotic resistance and its association with CDI among physicians, thus highlighting the need for increased awareness and surveillance of CDI in Qatar and characterizing the circulating strains.

The aim of the present study is to investigate the epidemiology of CDI in patients admitted to Hamad General Hospital and Al-Khor Hospital in Qatar and identify the specific ribotypes associated with CDI in Qatar. In addition, the influence of age and correlation with other factors such as proton pump inhibitor use, antibiotic use, existence of chronic conditions, etc was also investigated.

## Methods

### Patients

From October 2011 to August 2012, consecutive patients with suspected CDI admitted to Hamad General Hospital and Al-Khor Hospital, Qatar, were included in the study. Both of these hospitals are part of the Hamad Medical Corporation, an academic institute, which comprises seven hospitals and a total of 2,070 beds. Hamad General Hospital is a 603-bed hospital, serving the population of Doha city. Its services include trauma & emergency medicine, paediatrics, critical care, specialised surgery, specialised medicine, laboratory medicine and radiology services, The hospital treats between 1,200 and 1,500 patients on average per day. Al-Khor Hospital is a 149-bed community hospital serving Northern Qatar. The hospital services include emergency medicine, general surgery, orthopedics, ENT, urology, dentistry, endocrine medicine, gastroenterology, dialysis, psychiatry, paediatric medicine, obstetrics and gynecology and general intensive care.

Recruited patients included those that were admitted to the hospital with diarrhoea and those who developed diarrhoea during hospitalisation. The inclusion criteria were: presence of diarrhoea (watery, loose or unformed stool passed at a frequency of three times or more within 24 hours); recent (within the last 3 months) antibiotic exposure (as recent antibiotics exposure has been linked to increased risk of CDI); abdominal pain; fever; and a distinctive foul stool odour. Exclusion criteria included: Children ≤12 months of age, no recent (within 3 months) antibiotic exposure; gastrointestinal symptoms without diarrhoea. Patients were not included in the study more than once if they had recurrent symptoms.

Hospital acquired diarrhoea was defined as diarrhoea which developed ≥48 hours after admission. Community-acquired diarrhoea was defined as diarrhoea, which developed before 48 hours of admission. CDI was defined as diarrhoea in a patient with a stool culture positive for *C. difficile*.

The Research Ethical Committee of Hamad Medical Corporation, Qatar approved the study. Informed consent was obtained from all participants (or parents in case of children under 16 yr of age).

For the patients identified as CDI positive, risk factor analysis for association with specific ribotypes was carried out. Factors investigated for correlation with CDI included; proton pump inhibitors (PPI) use; older age; medicinal steroid use; non-steroidal anti-inflammatory drug (NSAID) use; immunosuppression; chemotherapy; chronic kidney disease (CKD);chronic liver disease (CLD); organ transplant; or inflammatory bowel disease (IBD) . These factors were primarily identified through patient’s medical history.

### Assessment & isolation of C. difficile

The stool samples were sent to the Hamad General Hospital Laboratory within a few hours of onset of diarrhoea.

The samples were tested for *C.difficile* using Glutamate Dehydrogenase (GDH) lateral flow assay (*Clostridium difficile* Quik Chek Complete, Techlab, Alere North America LLC), toxins A and B by Enzyme Immunoassay (EIA) and PCR as per the manufacturer’s instructions (GeneXpert, Cepheid, CA, USA).

GDH assay, toxins A and B EIA were first-line screening tests. All samples were then tested using PCR. If the PCR did not show consistent results with the initial screening tests during the first attempt, it was repeated using new reagents and a new cartridge. If the results were negative upon re-testing, the specimen was not considered *C.difficile p*ositive. Isolates that were shown to be *C. difficile* negative during the initial screen were not characterised further.

### DNA Extraction

*C. difficile* DNA was extracted from blood agar sub-cultured colonies, using a Chelex resin-based DNA extraction kit (InstaGene Matrix; Bio-Rad, USA).

### PCR Ribotyping

The *C.difficile* positive samples identified were cultured in *C.difficile* selective media (Cycloserine Cefoxitin Fructose Agar, CCFA) (bioMérieux, St. Louis, MO) after ethanol shock.

Each specimen (1 ml) was homogenized in 1 ml of absolute alcohol, vortexed to form an even suspension, and left to stand at room temperature for up to 1 hr. A 50-75 μl aliquot of this deposit was inoculated onto each of the Cycloserine _cefoxitin fructose plate agar to obtain isolated bacterial colonies. All media were incubated at 37°C in an anaerobic workstation. The cultures were incubated for 48 to 72 h. Tests carried out to positively identify C. difficile were the following; morphology, odour (Horse manure odour), fluorescence and latex (when necessary).

PCR-ribotyping was performed on the cultured pure colonies of *C. difficile* using the ribotyping protocol provided by Cambridge University, HPA Microbiology Services, Clinical Microbiology Laboratory, UK.

Briefly, amplification reactions were performed as per Leeds Laboratory Protocol [13], in 45 μl PCR tubes, containing 34.5 μl RNase/DNase free water; 5.0 μl of 10× RT-PCR reaction Mix; 2.25 μl mM MgCl_2_; 2 μl of 10 mM dNTP; 0.5 μl of 50 pmol/ul, forward and reverse primers for the intergenic 16S-23S rRNA Spacer Regions of *C. difficile* (Forward 5’- 6 FAM - GTG CGG CTG GAT CAC CTC CT- 3’ and reverse 5’- CCC TGC ACC CTT AAT AAC TTG ACC- 3’); 0.25 μl Taq polymerase; and 5 μl of extracted DNA template (or sterile water as a negative control). Amplifications were carried out in a thermal cycler (Touchgene Gradient; Techne) for 1 cycle of 2 min at 95°C for denaturation, followed by 30 cycles (1 min at 95°C, 1 min at 55°C and 1.5 min at 72°C) and a final extension 7 mins at 72°C.

### Fragment analysis

ABI-3130 (Applied Biosystem, USA) instrument with POP-gel was used for fragment analysis. Peak Scanner software v1.0 was used for data analysis. The peak sizes of all major peaks were compared with peaks from reference strains (34 reference strains from a library of the most commonly encountered ribotypes in the UK), allowing for deviations ±2 bp. The strains with banding patterns that did not match with the reference strains from the UK were sent to the Leeds Reference Laboratory, UK for ribotyping.

### Correlation analysis

Patient records were examined for factors potentially correlating with CDI (age; proton pump inhibitor (PPI) use; medicinal steroid use; NSAID use; immunosuppression; chemotherapy; chronic kidney disease; chronic liver disease; organ transplant; inflammatory bowel disease). Only records of CDI positive patients were examined. The correlation with ribotypes was investigated using Crosstab analysis.

## Results

### Patients

A total of 1,532 patients with suspected CDI were recruited. One hundred and twenty two stool samples, out of total 1,532 patients, were *C. difficile* positive and were selected for sample analysis (Table 1). Of the 122 CDI positive patients , 98 (80.3% or 6.4% of 1,532) had hospital acquired CDI and 14 (11.5% or 0.9% of 1,532) had community-acquired CDI. Medical records for the remaining 10 (8.2%) were not available to categorise them into hospital or community-acquired CDI. Amongst the hospital acquired patients, 5.7% (n = 87/1,532) were from Hamad General Hospital and 0.7% (n = 11/1,532) from Al Khor Hospital. Each sample represented an individual patient. All 122 patients were residents of Qatar though their ethnic background details are not available.

**Table 1:** **Baseline patient characteristics of 122 patients with CDI**

Variable	Category	No. Of subjects n (%)
Total number of subjects	Qatari residents	122
Gender	Males	72 (59%)
	Females	50 (41%)
Age	1-14 yrs	23 (18.9%)
	15-30 yrs	15 (12.3%)
	31-50 yrs	21 (17.2%)
	51-64 yrs	22 (18%)
	≥ 65 yrs	41 (33.6%)
Hospital acquired CDI		98 (80.3%)
Community acquired CDI		14 (11.5%)
Medical Records not available		10 (8.2%)
Antibiotics used prior to CDI (some patients were on >1):	Tazocin	55 (45.1%)
	Third generation Cephalosporins	50 (41%)
	Amoxicillin	22 (18%)
	Meropenom	20 (16.4%)
	Ciprofloxacin	20 (16.4%)
	Vancomycin	15 (12.3%)
	Co-trimoxazole	10 (8.2%)
	Azithromycin	9 (9.8%)
	Amikacin	6 (7.4%)
	Teicoplanin	4 (3.3%)
	Nitrofurantoin	2 (1.6%)
	Doxycyclin	2 (1.6%)
	Tigecyclin	1 (0.8%)
	Linezolid	1 (0.8%)
	Cloxacillin	1 (0.8%)
	Colistin	1(0.8%)
	Moxifloxacin,	1 (0.8%)
	Ampicillin	1 (0.8%)
	Septrin	1 (0.8%)
	Amygdalin	1 (0.8%)

### PCR ribotyping

Pure colonies of *C. difficile* were obtained from only 79 out of 122 CDI positive patient samples. In total, 36 different PCR ribotypes (Table 2) were identified among *C. difficile* strains isolated from the 79 successfully cultured strains. Of the 36 *C. difficile* PCR ribotypes isolated, the most frequent ones were 258 (n = 6 [7.6%]), followed by 001/014/046 (n = 5 [6.3%]), and then 011/053/056/107 (n = 4 [5%]), followed by other PCR ribotypes (51.9%) detailed in Table 2. The prevalence of the PCR-ribotype 027 was only 1.3% (n = 1). Hence, the PCR ribotypes predominantly found in Qatar during the period of this study were 258 followed by 001/014/046. Only one ribotype was isolated from each *C. difficile* colony.

**Table 2:** **Frequency and percentages of** ***C. difficile*** **ribotypes isolated in Qatar from 2011 to 2012 (n = 79)**

*C. difficile*ribotype	Frequency N (%)
258	6 (7.6%)
001	5 (6.3%)
014	5 (6.3%)
046	5 (6.3%)
011	4 (5.1%)
053	4 (5.1%)
056	4 (5.1%)
107	4 (5.1%)
015	3 (3.8%)
017	3 (3.8%)
023	3 (3.8%)
183	3 (3.8%)
016	2 (2.5%)
081	2 (2.5%)
084	2 (2.5%)
087	2 (2.5%)
097	2 (2.5%)
103	2 (2.5%)
104	2 (2.5%)
003	1 (1.3%)
004	1 (1.3%)
020	1 (1.3%)
027	1 (1.3%)
078	1 (1.3%)
106	1 (1.3%)
116	1 (1.3%)
126	1 (1.3%)
174	1 (1.3%)
186	1 (1.3%)
193	1 (1.3%)
216	1 (1.3%)
248	1 (1.3%)
353	1 (1.3%)
436	1 (1.3%)
095	1 (1.3%)

### Correlating factors with CDI

#### Age

The frequency of CDI in the 122 patients by age groups is shown in Table 1. The frequency of ribotypes isolated, from highest to lowest, was: 19 (24%) from the ≥ 65 yr group; 12 (15%) from the 1-14 yrs group; 12 (15%) from 51-64 yr group; 10 (12.6%) from the 15-30 yr group; and 9 (11.3%) from the 31-50 yr group. Both the CDI infection rate and frequency of ribotypes isolated were highest in the elderly age group (≥65 yr) (Table 1 for CDI).

#### Other correlates

Table 3 summarises other factors investigated for correlation with CDI. Several factors were thought to correlate with CDI, especially use of PPI treatment. Indeed, PPI use was more common in patients with CDI than being ≥65 yr of age. However, due to the small sample sizes, statistical tests couldn’t be accurately performed on this data.

**Table 3:** **Factors investigated for correlation with CDI (n = 122)**

Correlating factors	Infected with CDI N = 122 (%)	Correlating ribotypes
PPI use*^†^	65 (53.3%)	014, 258, 046,053, 017 and 001
Age ≥ 65 yr	41 (33.6%)	107, 001,103, 011,116, 126,015, 017, 183, 186, 003,046, 053, 056, 078, 081, 084
Medicinal steroid use*^†^	29 (23.7%)	258, 014, 001,106, 107, 015, 016, 183, 193, 353, 046, 081, 095
NSAID*^†^	28 (23%)	107, 258, 011, 103, 014, 174, 183,193, 020, 353, 004, 046, 056, 081, 084, 087
Immunosupression	25 (20.5%)	014, 015, 046 , 258, 001,106, 116, 016,017, 183, 186, 193, 353
Chemotherapy*^†^	18 (14.7%)	046, 001, 106, 116, 014,183,186, 193, 216, 258,003, 353
CKD	17 (14%)	011, 116,126, 014, 017, 174, 046, 078, 084
CLD	5 (4.1%)	183, 186, 003
Organ-transplant	4 (3.3%)	011
IBD	1 (0.8%)	015, 056

*PPI: Proton Pump Inhibitor; *NSAID*: Non-steroidal anti-inflammatory drug; *IBD*: Inflammatory bowel disease; *CKD*: Chronic kidney disease; *CLD*: Chronic liver disease.

^†^All treatments were administered up to 3 months prior to acquiring CDI.

*NS*: Non-significant.

## Discussion

Our study showed presence of CDI in 122 of the 1,532 patients (prevalence of 7.9% during the period 2011 to 2012; 6.4% of which were hospital acquired). Amongst the hospital acquired patients, 5.7% (n = 87/1,532) were from Hamad General Hospital and 0.7% (n = 11/1,532) from Al Khor Hospital. The reason for the large difference between the hospitals is probably due to the presence of more patient samples, in our study, from Hamad General Hospital vs. Al Khor Hospital. Of the 122 CDI positive patients 98 (80.3%) had hospital acquired CDI and 14 (11.8%) had community-acquired CDI. The prevalence data found in our study are comparable to data from other Middle-Eastern countries (prevalence range 4.6-13.7%; average ~8.6%) [14–18]. Comparatively, in Europe *C. difficile* infections are responsible for only 3.6% of all hospital acquired infections [19]. The difference may be attributed to over-prescription of antibiotics in the Middle East. However, it must be noted that the sample sizes used in this study and in the cited Middle Eastern studies are quite small compared to the large surveillance data gathered by the European surveillance study [19]. The key finding is that CDI appears to be doubled in patients in hospitals in the Middle East compared with Europe, and may be under-diagnosed . The difference in CDI illustrates the importance of active surveillance of CDI in hospitals in the Middle East and a need for regulation of antibiotic prescription.

The PCR ribotypes predominant in Qatar during this period were 258 followed by 001/014/046. The most common ribotypes isolated from two other countries in the Middle East were: 002, 001, 126 and 140 from Kuwait [16]; and 078 from Iran [20], respectively. However, there is very little data on other specific riborypes in the Middle East; whereas ribotypes 017, 018, 014, 002 and 001 have been shown to be most prevalent in Asia [21–25].

A study carried out by Bauer and colleagues in 34 European countries identified PCR ribotypes 014/020, 001, and 078 as the most prevalent in Europe [26]. The prevalence of PCR-ribotype 027 was only 5% in this study [26], although this ribotype had been previously associated with increasing outbreaks of CDI in hospitals in North America, Japan and Europe [27]. This ribotype was isolated from only one sample in the present study and was not isolated in either the Kuwaiti or Iranian studies mentioned earlier [16, 20]. It is important to note here that we managed to culture only 79 samples successfully out of the 122 with CDI. This low number might have led to under-representation of PCR ribotypes that caused CDI in our patient population.

The antibiotics most frequently correlating with CDI in our study were third generation cephalosporins and piperacillin/tazobactam. Third generation cephalosporins, even when administered as short-term perioperative prophylaxis, have been significantly associated with *C. difficile*-related diseases [28, 29]. Piperacillin/tazobactam has also been linked to development of CDI, though tazocin is generally associated with lower risk of *C. difficile* infections [30, 31]. The correlation of CDI with piperacillin/tazobactam in our study may reflect the over-prescribing of antibiotics in Qatar. The use of certain antibiotics, especially fluoroquinolones, has been previously associated with infection by PCR-ribotype 027 [27, 32]. In our study the ribotype most associated with third generation cephalosporins and piperacillin/tazobactam use was 258 in 6 patients (n = 2 and n = 4 for each antibiotic, respectively).

In our study, CDI was most often correlated with older age (≥65 yrs) and use of PPIs. However, it must be noted that the number of infections for which these correlations were based was small. Old age and PPIs have been previously reported to be risk factor for CDI [33–35]. Controlled prescribing of PPIs, especially in high risk individuals, may be important in control of CDIs. No ribotype correlated significantly with any risk factor investigated.

Unfortunately patient outcomes after confirmation of CDI were not investigated, which is a limitation of this study and needs to be addressed in future studies. Additional limitations include missing data for the 10 patients, which could not be classified in hospital-acquired or community-acquired CDI. These patients comprise 8.2% of the CDI positive population and their inclusion would have been useful here. Furthermore, in this study, we had small sample sizes for each age group and hence correlation of CDI with age will probably have to be repeated with larger samples. Overall, Qatar population is ~350,000 people and, although a relatively homogenous population, recruiting for clinical studies is limited by the small population size. Finally, while ribotype 027 was only identified in one sample, further surveillance is required to determine whether this strain is indeed present in the country and to monitor for its prevalence in a larger sample set.

## Conclusion

The commonest *C. difficile* ribotype identified in Qatar was different from those found in North America, Europe and Asia. In addition, the prevalence of CDI was higher in Qatar than Europe; though comparable to other Middle Eastern countries. Restrictions on the use of antibiotics and PPIs should be considered in the Middle East. Unrestricted prescribing of medicine is complicated further by a lack of epidemiology data and surveillance of CDI in Middle-Eastern countries. These findings underscore the importance of local surveillance to detect and control *C. difficile* infection. The findings also highlight a need to control PPI prescriptions, especially in the elderly population.

## Authors’ original submitted files for images

Below are the links to the authors’ original submitted files for images. Authors’ original file for figure 1
