# Erratum: Polymerase chain reaction ribotyping of Clostridium difficile isolates in Qatar: a hospital-based study

**DOI:** 10.1186/s12879-015-0866-3

**Published:** 2015-04-03

**Authors:** Asma A Al-Thani, Wedad S Hamdi, Naser A Al-Ansari, Sanjay H Doiphode, Godwin Justus Wilson

**Affiliations:** Health Sciences Department, College of Arts and Sciences, Biomedical Research Center, Qatar University, Doha, Qatar; Department of Laboratory medicine and Pathology, Al-Khor Hospital, Hamad Medical Corporation, Doha, Qatar

Following publication of this article [1] we noted that we had made an error in the authorship list. The correct details can be found as above.

The authors contributions section should be updated as follows:

A.Al-T. designed this study. Wedad Saleem performed data analysis and drafted the manuscript. N. Al-A. provided assistance with data collection and manuscript revision. S.D. collected samples and clinical data, participated in the design of the study and performed the statistical analysis. G.W. was involved in the concept and design of the study and he analysed the data. All authors read and approved the final manuscript.
