# Supplementary figures and images for: Polymerase chain reaction ribotyping of Clostridium difficileisolates in Qatar: a hospital-based study
Source: BMC Infect Dis. 2014 Sep 15;14:502. doi: 10.1186/1471-2334-14-502 (PMC4262129; doi:10.1186/1471-2334-14-502)

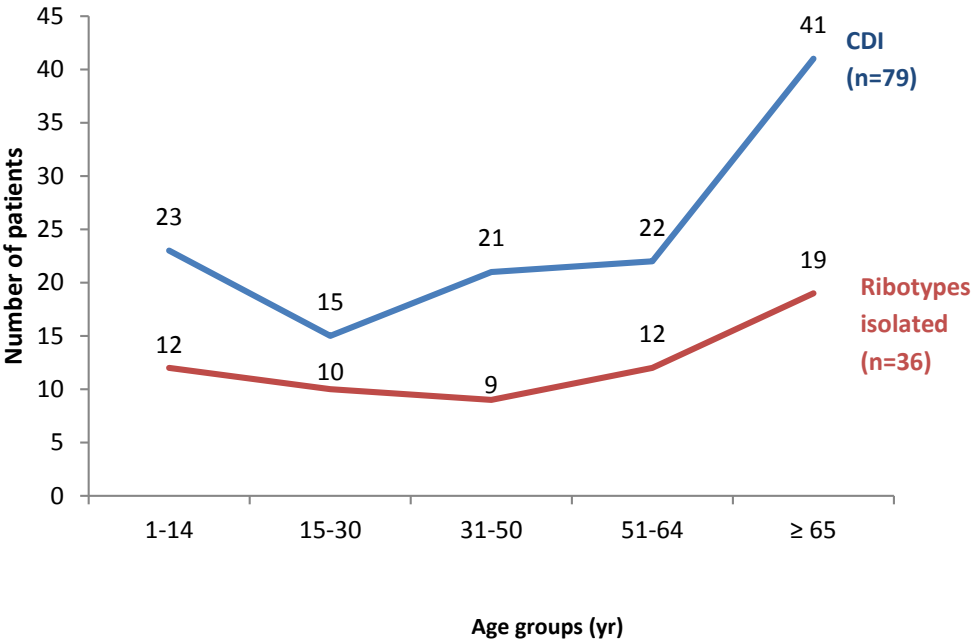

Supplement: Supplementary file 1 — Authors’ original file for figure 1 [file 12879_2014_4049_MOESM1_ESM.pdf]
